# Catheter-Related Right Atrial Thrombus Requiring Surgical Embolectomy

**DOI:** 10.7759/cureus.17641

**Published:** 2021-09-01

**Authors:** Deborah T Akanya, Jay Parekh, Soniya Abraham, Sam Uche, Gilead Lancaster

**Affiliations:** 1 Cardiology, Yale New Haven Health/Bridgeport Hospital, Bridgeport, USA; 2 Internal Medicine, Yale New Haven Health/Bridgeport Hospital, Bridgeport, USA; 3 Data Analyst/Health Information Technologist, Cigna Health Insurance, Bloomfield, USA

**Keywords:** atrium thrombus, right heart cath, end stage renal disease (esrd), pulmomary embolus, echo cardiogram

## Abstract

Temporary central venous hemodialysis (HD) catheters are commonly used in end-stage renal disease (ESRD) patients while awaiting peritoneal dialysis catheter, arterio-venous fistula or graft placement and maturation. Catheter-related right atrial thrombus (CRAT) is a common finding in patients with central venous catheters (CVCs) and can cause CVC to malfunction. The incidence of CRAT is about 29% with a mortality of 18.3% or greater if not identified and managed appropriately. Two major types of right atrial (RA) thrombi have been identified. Type A thrombus usually originates in the peripheral veins embolizing to the RA. Type B originates within a structurally abnormal RA and is usually attached to the chamber walls or foreign bodies like CVC or intra-cardiac wires. There is a high risk of thrombi embolization leading to pulmonary embolism as in our patient, systemic embolization if a right to left shunt is present and potential hemodynamic compromise. The optimal therapeutic approach is still a subject of discussion, but timely catheter removal with prompt initiation of systemic anticoagulation is key. Surgical management is pursued when medical therapy fails or if the thrombus is greater than 6 cm. Our case is that of a 30-year-old male with CRAT successfully treated with surgical embolectomy after the failure of systemic anticoagulation. This case highlights the importance of early detection of CRAT, initiation of optimal medical therapy and the need for surgical intervention when medical therapy fails.

## Introduction

Catheter-related right atrial thrombus (CRAT) is usually an incidental finding but with the increase in the use of central venous catheters (CVCs), the incidence of CRAT is rising. As clinicians, we should have a high index of suspicion of CRAT when a CVC malfunctions. A malfunctioning CVC may present with resistance in blood flow, this may represent an early sign of CRAT. Our paper reviews published literature as well as highlights the importance of a multidisciplinary approach in the management of CRAT, the role of prompt catheter removal, systemic anticoagulation and eventual surgical removal which are key in reducing the associated mortality from this entity [1].

## Case presentation

**Figure 1:**
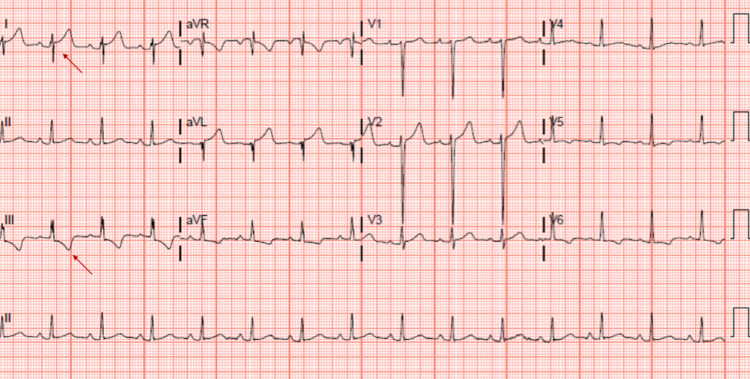
Electrocardiogram showing normal sinus rhythm, rightward axis, S1T3 (red arrows) and left ventricular hypertrophy by voltage criteria.

A 30-year-old male presented to the emergency department (ED) post hemodialysis (HD) for evaluation of positive blood cultures. His medical history was significant for hypertension, type 1 diabetes mellitus and end-stage renal disease (ESRD) secondary to diabetic nephropathy. Four days prior to his presentation, he was noted to have profuse drainage around his tunneled dialysis catheter site and blood cultures were obtained. The blood culture gram stain showed budding yeast with pseudo-hyphae, and he was referred to the ED. He endorsed a three-day history of lethargy, nausea, vomiting, poor oral intake and seeing translucent black lines in his left eye. He was afebrile on presentation to the ED, his blood pressure was 183/117 mmHg, heart rate of 99 beats/min and saturating 100% on room air. Laboratory data were significant for eosinophilia of 7.3% ( 0.4%-4.0%) with an absolute eosinophil count of 0.7 x 1,000/µL (0.0-0.4 x 1,000/µL). Electrocardiogram (ECG) showed normal sinus rhythm, and a new rightward axis with S-wave in lead I, T-wave inversions in lead III and the lateral leads (Figure 1).

 

These changes were concerning for pulmonary embolism (PE). Chest computed tomographic angiography showed filling defects in the right segmental and sub-segmental pulmonary arteries. Transthoracic echocardiography (TTE) to evaluate for vegetations showed a normal left ventricular ejection fraction and moderate concentric left ventricular hypertrophy. There was a 3.3 cm x 2.5 cm echo-density seen in the right atrium (RA) and moderate pericardial effusion without echocardiographic evidence of tamponade (Video 1).

**Video 1:** Apical four-chamber standard TTE view showing an echo-density in the RA. TTE - Transthoracic echocardiography; RA - right atrium

A transesophageal echocardiogram (TEE) was performed to further characterize the RA mass. It showed a 3.2 cm x 2.4 cm partially mobile mass attached to the RA free wall with a large sessile mural component extending in the superior vena cava (SVC). There was a partially occlusive mass in the SVC (Videos 2-4).

**Video 2:** Standard mid esophageal TEE view showing a partially mobile mass in the RA. TEE - transesophageal echocardiogram, RA - right atrium

**Video 3:** Bicaval TEE view showing RA and SVC masses. TEE - transesophageal echocardiogram, RA - right atrium; SVC - superior vena cava

**Video 4:** X-plane of the bicaval view showing SVC mass. SVC - superior vena cava

**Figure 2:**
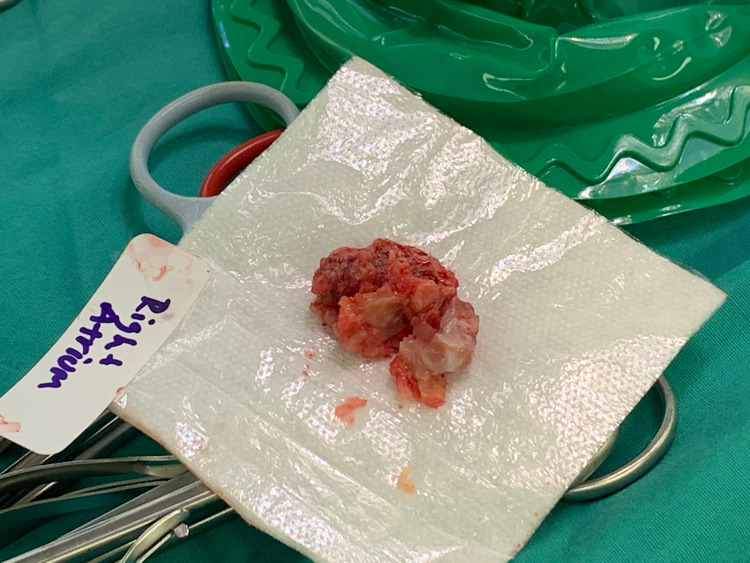
Gross pathology excised RA mass. RA – right atrium

**Figure 3:**
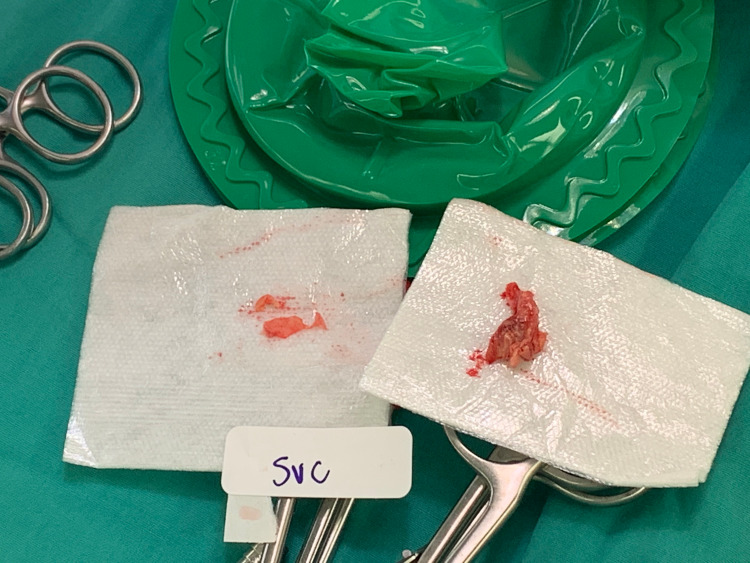
Gross pathology of excised SVC mass. SVC – superior vena cava

There was no shunt across the interatrial septum on doppler or with agitated saline and no evidence of mass or vegetation on any other cardiac structures. As the likely source of his fungemia was his CVC, interventional radiology was consulted for catheter removal and peritoneal dialysis was initiated. Anidulafungin was initiated as per infectious disease recommendations and later de-escalated to oral fluconazole after blood cultures speciated as Candida Albicans. Candida endophthalmitis was ruled out by ophthalmology. Systemic anticoagulation with IV Heparin was initiated for PE and suspected RA thrombus. Repeat TTE a couple of days later showed persistence with interval increase in the size of the RA mass and survival excision was recommended by cardiothoracic surgery. Intra-operative inspection of the RA showed a soft gelatinous mass with a narrow base between the fossa ovalis and coronary sinus. Inspection of the SVC revealed a separate sessile mass on a wide stalk near the SVC by-pass cannulation site. Both masses were excised, the RA and SVC were reconstructed with a bovine pericardial patch (Figures 2, 3).

**Figure 4:**
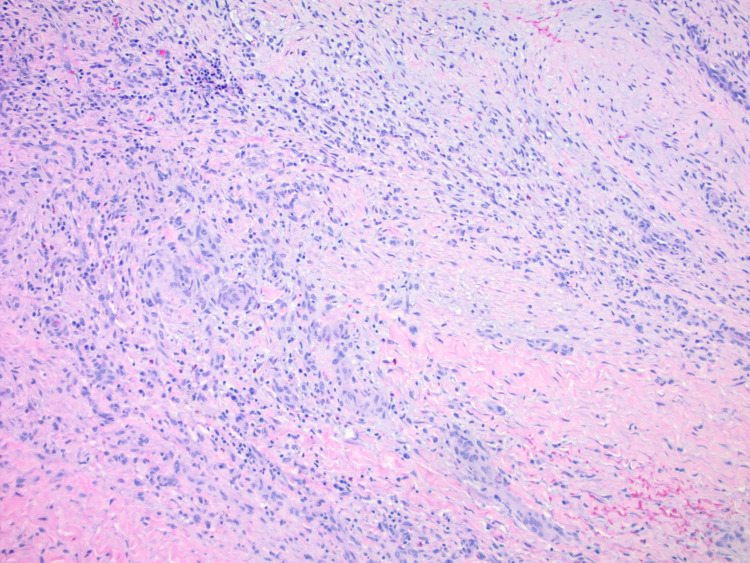
Laminated RA thrombus with focal granulation. RA – right atrium

**Figure 5:**
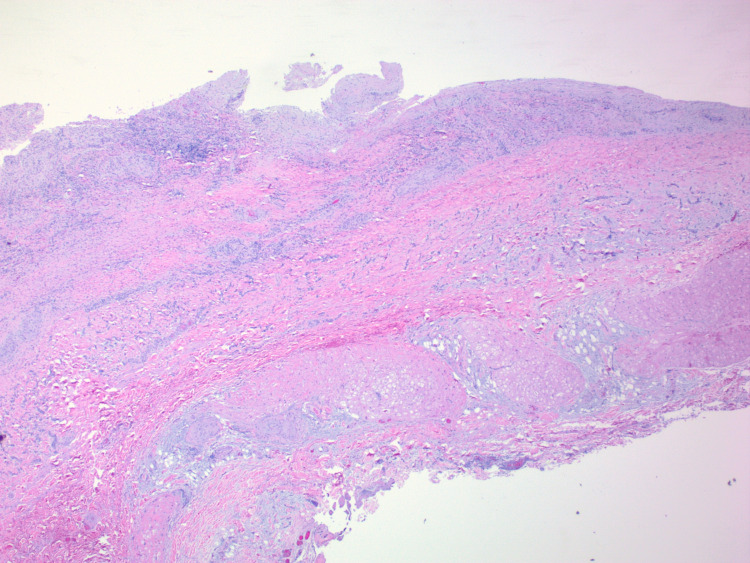
Focal vasculitis of the SVC. SVC – superior vena cava

Histopathology confirmed both masses to be laminated thrombi with no apparent micro-organisms or malignant cells (Figure 4). There was focal vasculitis of the SVC (Figure 5).

Intra-op TEE showed no residual masses. His immediate postoperative period was complicated by left side pneumothorax managed with chest tube placement. He was discharged on hospital day 17 with apixaban and fluconazole. At his one-month follow-up, he had no post-surgical or bleeding complications. He completed six weeks of fluconazole and six months of systemic anticoagulation with apixaban.

## Discussion

Temporary central venous HD catheters are used in ESRD patients while awaiting peritoneal dialysis catheter, arterio-venous fistula or graft placement and maturation. In our patient, a central venous HD catheter was placed for a nonfunctional arterio-venous fistula. CRAT is a common finding in patients with CVC, the incidence is about 29% [1] with a mortality of 18.3% [2]. CRAT can be an incidental finding as in our case but should be suspected when CVC malfunctions. Two major types of RA thrombi have been identified. Type A thrombus is the most common, it is elongated, worm-like in appearance as it mostly originates from the peripheral veins in the setting of deep vein thrombosis. Type A thrombus can be seen in transit straddling a patent foramen ovale. Type B thrombus as in our patient is oval, it originates within a structurally abnormal RA and is usually attached to the chamber wall or foreign bodies like CVC or intracardiac wires.

CRAT results from mechanical irritation of the RA wall by the catheter tip movement. This continuous irritation results in endothelial damage, platelet aggregation and activation of the clotting cascade resulting in thrombus formation. Also, hypercoagulability in ESRD patients is thought to contribute to thrombi formation [3]. Both types of thrombi can embolize, but type A thrombi have a higher rate of embolization as well as higher mortality. Patients with CRAT may be asymptomatic or develop symptoms from complications like PE, systemic embolism if a right to left shunt is present or hemodynamic compromise from right-sided heart failure. TEE has higher sensitivity and specificity than TTE in making this diagnosis. Cardiac computed tomographic imaging and cardiac magnetic resonance imaging with contrast both help with tissue characterization and establishing this diagnosis.

The optimal therapeutic approach is still a subject of discussion as no randomized controlled trials have directly compared the available treatment methods, but early catheter removal is essential as it allows for spontaneous resolution of the thrombus in some cases. Systemic anticoagulation prior to CVC removal is recommended due to concerns for embolization during catheter manipulation. Systemic thrombolysis has been used in cases complicated by massive PE or if anticoagulation fails [4]. When anticoagulation is effective it should be continued for at least six months or till the complete resolution of the thrombus.

Surgical embolectomy is the treatment of choice when anticoagulation is contraindicated, the thrombus fails to resolve, for thrombus > 6 cm, or cardiac abnormalities or endocarditis with an indication for surgery. Studies have shown no mortality benefits between patients treated with systemic anticoagulation versus surgical embolectomy. Suction thrombectomy can be considered in high-risk patients but data regarding its utility are still scarce.

## Conclusions

The histopathology findings of laminated thrombi with no apparent micro-organisms suggest that the pathophysiology of our patients' RA mass was not infectious but related to continuous mechanical irritation of the RA by his CVC. Echocardiography played a role in the early detection of his type B CRAT. A multidisciplinary approach with prompt catheter removal, initiation of antifungals, systemic anticoagulation, eventual surgical resection were key in his management and reducing the associated mortality of his CRAT. Clinicians should therefore keep a broad view of differentials in sick patients with CVCs, as well as have a high index of suspicion of CRAT when CVC malfunctions.
